# Coinfection pulmonaire par *pneumocystis jirovecii* et *pseudomonas aeruginosa* au cours du SIDA: à propos de deux cas

**DOI:** 10.11604/pamj.2015.21.95.5993

**Published:** 2015-06-05

**Authors:** Savadogo Mamoudou, Guillaume Bellaud, Canestri Ana, Pialoux Gilles

**Affiliations:** 1 Service des Maladies Infectieuses du CHU Yalgado Ouédraogo, Ouagadougou Burkina Faso; 2 Service des Maladies Infectieuses, Hôpital Ténon, Paris, France

**Keywords:** coinfection, Pneumocystis jirovecii, Pseudomonas aeruginosa, pneumopathie, coinfection, Pneumocystis jirovecii, Pseudomonas aeruginosa, pneumopathy

## Abstract

Rapporter deux cas cliniques de coinfections pulmonaires par *Pneumocystis jirovecii* et par *Pseudomonas aeruginosa* chez des patients vivant avec le VIH. Les deux patients étaient âgés respectivement de 32 ans et 46 ans. Un patient a été pris en charge à l'hôpital Yalgado Ouédraogo de Ouagadougou au Burkina Faso et l'autre a été pris en charge à l'hôpital Ténon de Paris, en France. Les deux souffraient de pneumopathie confirmée à la radiographie et à la tomodensitométrie. L'un des patients était sévèrement immuno déprimé, contrairement à l'autre. L'examen bactériologique dans les crachats avait permis d'isoler *Pseudomonas aeruginosa* et *Pneumocystis jirovecii* chez les deux patients. Sous traitement, l’évolution a été favorable. Les coinfections morbides sont relativement fréquentes chez les patients vivant avec le VIH. Devant une symptomatologie respiratoire du sujet vivant avec le VIH, il faut savoir rechercher en plus du Bacille de Koch, *Pneumocystis jirovecii* et *Pseudomonas aeruginosa* par un lavage broncho alvéolaire.

## Introduction

Plus de trente ans après la découverte du virus de l'immunodéficience humaine (VIH) et plus de dix-sept ans après l'arrivée des antirétroviraux puissants, les infections respiratoires opportunistes demeurent des complications fréquentes des patients infectés par le VIH [1]. Avant l'avènement du traitement antirétroviral, leur prévalence atteignait 80% chez les patients vivant avec le VIH au stade Sida. Mais depuis l'arrivée des ARV puissants, leur incidence a considérablement diminuée [2]. Si ces infections restent plus fréquentes chez les patients sévèrement immunodéprimés [1], elles peuvent aussi survenir sur terrain immunocompétent [3]. Nous rapportons deux cas de coinfections pulmonaires opportunistes chez des patients vivant avec le VIH au stade SIDA.

## Patient et observation

### Observation 1

Une veuve de 32 ans a été dépistée positive au VIH1 au décours d'une perte pondérale d'installation progressive. Sur le plan fonctionnel, elle présentait une anorexie et une toux productive modérée qui évoluerait depuis plus de deux semaines. L'examen clinique notait un assez bon état général, des conjonctives bien colorées anictériques, un poids= 46kg, une TA= 110mmHg/70mmHg, une température à 37^°^8C, un pouls= 80bat/mn, la FR = 20 cycles/mn, la taille= 1,60m, IMC= 18kg/m^2^. L'examen de l'appareil respiratoire notait des râles crépitant bilatéraux, plus marqués au champ pulmonaire gauche. Le reste de l'examen clinique était sans particularité. L'examen biologique notait une anémie avec 8,5 g/dl, le dosage des lymphocytes TCD4 était revenu avec 750 cellules/mm^3^ et il n'y avait pas d'anomalie des fonctions hépatiques et rénales. La charge virale n'avait pas pu être réalisée. L'examen des crachats à la recherche des BAAR est revenu négatif et la radiographie pulmonaire notait une pneumopathie interstitielle bilatérale. Mise sous antibiothérapie à base d'amoxicilline + acide clavulanique puis sous spiramycine, l’évolution a été marquée par la persistance des signes malgré deux semaines de traitement. Une fibroscopie bronchique avec lavage broncho alvéolaire(LBA) demandée a permis d'isoler Pseudomonas aeruginosa sensible à l'amikacine, à la ceftazidime, à la ciprofloxacine, à la gentamicine, à l'ofloxacine, à la pipéracilline/tazobactam, à la ticarcilline et à la tobramycine. Elle était résistante à l'acide nalidixique, à l'association amoxicilline + acide clavulanique, à l'ampicilline, à la céfalotine, à la céfoxitine, et à la nitrofurantoïne. L'examen mycologique du pu avait par ailleurs isolé à l'examen direct et après coloration au MGG la présence de formes trophiques de *Pneumocystis jirovecii*. Sous traitement à base de Nethilmicine 1Ampx2/j, Triméthoprime + Sulfamethoxazole forte 2cpx3/j pendant 21 jours, et sous Ténofovir + Lamivudine + Efavirenz, l’évolution a été favorable.

### Observation 2

Patient de 46 ans ayant des antécédents de broncho-pneumopathie chronique obstructive (BPCO) et de tabagisme, a été référé par son pneumologue au service des maladies infectieuses et tropicales (SMIT) de l'hôpital Ténon, pour pneumopathie de la base gauche. Le début de sa maladie remonterait à 6 mois environ marqué par une dyspnée d'installation progressive qui l'avait amenée à consulter son pneumologue. Une radiographie et un scanner pulmonaires prescrits par ce dernier, avaient permis de diagnostiquer une pneumopathie de la base gauche qui évoluait dans un contexte de fièvre à 40^°^C associée à une dyspnée et à une cyanose. Il a été admis au service des SMIT de l'Hôpital Ténon, pour prise en charge. L'examen clinique à son admission notait à l'examen général une asthénie, une anorexie, des conjonctives bien colorées anictériques, la tension artérielle était égal à 103/67 mmHg; la fréquence cardiaque à 115 battements/mn; la température était égal à 39,4^°^C. A l'examen de l'appareil respiratoire, le patient était sous oxygène. L'examen notait une dyspnée à type de polypnée avec une fréquence respiratoire à 38 cycles par minute, il n'avait pas de cyanose, ni de tirage. A l'auscultation le murmure vésiculaire était diminué aux deux champs pulmonaires. A l'examen de l'appareil cardiovasculaire, le thorax était de morphologie normale, il n'y avait pas de douleur thoracique à la palpation, il y avait une tachycardie avec une fréquence cardiaque à 115 battements/minute, il n'avait pas de signe d'insuffisance cardiaque, les pouls périphériques étaient bien perçus synchrones, les bruits du cœur étaient assourdis mais réguliers, il n'y avait pas de bruits surajoutés. L'examen de la peau notait une dermite séborrhéique. Le reste de l'examen clinique était sans particularité.

**Résultats du bilan complémentaire demandé:** la sérologie VIH est positive. Les lymphocytes TCD4 était à 21 cellules/mm^3^. La charge virale VIH1 était à 1000000 copies/ml. La recherche d'ADN de *Pneumocystis jirovecii* dans le crachat induit était positive. La recherche des BAAR dans les crachats était négative. L'antigénemie cryptococcique est revenue positive. La recherche du cryptocoque dans les crachats est revenue négative. L'examen bactériologique des crachats a permis d'isoler Pseudomonas aeruginosa. IgG anti CMV était positif. Le traitement institué était à base de: augmentin + rovamycine (augmentin 1000mgx2/j et rovamycine 3 MUI x3/j) pour la pneumopathie à Pseudomonas aeruginosa; ambisome(1mg/kg/j en IV), ancotil (100mg/kg/j) pendant 14 jours puis relais par fluconazole 400mg/j pour la cryptococcose; cotrimoxazole forte 4ampx3/J en IV, associé à une corticothérapie, et à une oxygénothérapie pour la Pneumocystose. Ce traitement a été relayé par un traitement d'entretien avec du cotrimoxazole Forte 1 cp/j. La mise sous ARV a été différée pour éviter un syndrome de restauration immunitaire. L’évolution a été favorable et il est sorti de l'hôpital avec un rendez pour l'initiation du traitement antirétroviral.

## Discussion

Les coinfections morbides sont relativement fréquentes chez les patients vivant avec le VIH au stade sida [4]. L'arrivée des combinaisons antirétrovirales puissantes, a permis de réduire la morbi-mortalité liée aux infections opportunistes au cours de l'infection par le VIH. Mais beaucoup de patients paient encore un lourd tribut à ces infections parce qu'ils sont dépistés tardivement [5]. Selon la virulence de l'agent pathogène, les infections opportunistes correspondantes peuvent apparaître plus ou moins tôt dans l’évolution de l'infection à VIH. Le dosage des lymphocytes TCD4 a fait sa preuve en tant que marqueur de l’état immunitaire au cours de l'infection à VIH. La connaissance du taux de lymphocytes TCD4 permettait d’évaluer le risque encouru par chaque patient vis-à-vis des infections opportunistes [6]. Ainsi la Pneumocystose et la Cryptococcose surviennent habituellement chez des patients VIH positif présentant un taux de lymphocytes T CD4 inférieur à 200 cellules/mm^3^ [5]. Mais ces infections peuvent également survenir chez des patients immunocompétents [2, 3]. Egalement, des cas de colonisations par *Pneumocystis jirovecii* ont été décrits dans la littérature [7], surtout chez des patients dont le taux de lymphocyte TCD4 était supérieur à 200/mm^3^. La pneumocystose est l'infection classique du SIDA [8]. Son incidence a explosé avec l’épidémie de l'infection à VIH. Ce fut d'ailleurs cette infection opportuniste qui a permis la première description du syndrome d'immunodéficience acquise (sida) [8]. Elle se manifeste par une toux sèche avec dyspnée d'installation progressive réalisant une pneumopathie interstitielle hypoxémiante souvent bilatérale évoluant dans un contexte fébrile [3, 6]. Cette symptomatologie se rapproche de celle due aux infections respiratoires à *Pseudomonas aeruginosa* où sont fréquemment rencontrées la fièvre, la dyspnée, et la toux [9]. *Pseudomonas aeruginosa* est une bactérie opportuniste capable d'infecter presque tous les sites anatomiques dont l'appareil respiratoire [1]. Cette bactérie colonise volontiers les patients immunodéprimés et elle est naturellement peu sensible aux antibiotiques [4]. Le processus infectieux se déroule habituellement en trois étapes: adhésion et colonisation, invasion locale, puis dissémination et infection systémique au cours de laquelle l'atteinte pulmonaire peut s'observer [9]. Le diagnostic de ces infections respiratoires doit être suspecté devant un échec d'une antibiothérapie non spécifique et/ou devant une recherche BAAR négative dans les crachats. L'analyse des crachats induits ou à défaut, la fibroscopie avec lavage broncho alvéolaire (LBA) offre une sensibilité élevée. Le LBA et la PCR des crachats ont permis d'isoler *Pneumocystis jirovecii* [3] chez nos deux patients. Si le LBA est préconisé par plusieurs auteurs pour le diagnostic de la pneumocystose [3, 8], d'autres par contre préfèrent la Polymerase Chain Reaction (PCR). Mais une PCR à *Pneumocystis jirovecii* positive ne signifie pas toujours l'existence d'une infection patente et pourrait traduire une colonisation [3, 7, 8]. Il en est de même pour le dosage sérique du β-D-glucan qui peut être positif aussi bien en cas d'infection par *Pneumocystis jirovecii* que lors d'autres infections fongiques comme l'aspergillose et la candidose [8]. L'antigénemiecryptococcique était positive chez un des patients, comme chez environ 40% des patients séropositifs pour le VIH et chez 29% des sujets séronégatifs de certaines séries françaises [2]. Le traitement de référence de la pneumocystose fait appel, en absence de contre-indication au Triméthoprime + Sulfamethoxazole par voie orale ou intraveineuse à la dose de 75 mg à 100mg/kg/j pendant trois semaines. Cette molécule peut être remplacée par l'atovaquone ou de la pentamidine intraveineuse en cas d'effets secondaires graves liés au cotrimoxazole [2, 5, 10]. Les associations Triméthoprime + Dapsone et Clindamycine + Primaquine sont utilisées en deuxième ligne de traitement et sont réservées aux formes peu sévères de la pneumocystose [10]. La corticothérapie par voie générale est indiquée en cas d'hypoxie sévère. On utilise alors la prednisone à la dose de 1 mg/kg/j [3]. En cas d'hypoxémie sévère une oxygénothérapie est associée. Le traitement des infections à *Pseudomonas aeruginosa* repose sur un choix rationnel d'antibiotiques, et l'optimisation de leur usage sur des bases pharmacodynamiques [1]. Il sera le plus souvent une bithérapie choisie sur la base de l’épidémiologie locale (β-lactamines plus aminoglycoside ou fluoroquinolone) [10]. Ce traitement devra être réajusté sur la base des données de l'antibiogramme, et sur l’évolution clinique [1]. Quant au traitement de la Cryptococcose, il fait appel à l'amphotéricine B (0,7 à 1 mg kg/j) associé au 5-fluorocytosine (100 mg kg) pendant 15 Jours suivi d'un traitement d'entretien avec le fluconazole (400 mg/j) pendant 8 à 10 semaines [2].

## Conclusion

L'infection à VIH expose les personnes vivant avec le VIH sans traitement antirétroviral, aux infections opportunistes à localisation pulmonaire et systémiques. Devant une symptomatologie respiratoire du patient vivant avec le VIH, le diagnostic de pneumonie à *Pneumocystis jirovecii*, et à *Pseudomonas aeruginosa* doit être évoqué parmi les hypothèses diagnostiques, et activement recherché par un lavage broncho alvéolaire ou dans le crachat induit. Une bonne prise en charge de ces infections opportunistes est essentielle pour réduire la létalité des patients vivant avec le VIH. Enfin, il est indispensable de renforcer le dépistage de l'infection à VIH dans la population générale, afin de proposer une prise en charge qui permet d′éviter la survenue des pathologies opportunistes.
